# Targeted Tumor Therapy Remixed—An Update on the Use of Small-Molecule Drugs in Combination Therapies

**DOI:** 10.3390/cancers10060155

**Published:** 2018-05-24

**Authors:** Martina V. Gatzka

**Affiliations:** Department of Dermatology and Allergic Diseases, University of Ulm, 89081 Ulm, Germany; martina.gatzka@uni-ulm.de; Tel.: +49-731-500-57531

**Keywords:** apoptosis, clinical trial, DNA-damage response, epigenetics, kinase inhibitor, melanoma, metabolomics, small-molecule, targeted therapy, tumor

## Abstract

Over the last decade, the treatment of tumor patients has been revolutionized by the highly successful introduction of novel targeted therapies, in particular small-molecule kinase inhibitors and monoclonal antibodies, as well as by immunotherapies. Depending on the mutational status, BRAF and MEK inhibitor combinations or immune checkpoint inhibitors are current first-line treatments for metastatic melanoma. However, despite great improvements of survival rates limitations due to tumor heterogeneity, primary and acquired therapy resistance, immune evasion, and economical considerations will need to be overcome. Accordingly, ongoing clinical trials explore the individualized use of small-molecule drugs in new targeted therapy combinations based on patient parameters and tumor biopsies. With focus on melanoma therapy this review aims at providing a comprehensive overview of such novel alternative and combinational therapy strategies currently emerging from basic research. The molecular principles and drug classes that may hold promise for improved tumor therapy combination regimens including kinase inhibition, induction of apoptosis, DNA-damage response inhibition, epigenetic reprogramming, telomerase inhibition, redox modulation, metabolic reprogramming, proteasome inhibition, cancer stem cell transdifferentiation, immune cell signaling modulation, and others, are explained in brief. In addition, relevant targeted therapy combinations in current clinical trials and individualized treatment strategies are highlighted.

## 1. Introduction

Cancer pathogenesis evolves like a drama with a series of acts—including tumor initiation, promotion, progression, invasion, immune evasion, metastasis, chemo-resistance, and further progression ([1,2,3] and others). As per the model of multi-step tumorigenesis, mutations in oncogenes and in tumor suppressor genes as well as further genetic and epigenetic changes mediate this transformation process [1,2]. In interaction with the tumor micro-environment tumor cell plasticity and clonal evolution decide about tumor cell survival and aggressiveness-leading to substantial heterogeneity within the tumor as major limitation to durable therapeutic responses [4]. Conventional cytotoxic drugs (“chemotherapy”) for the treatment of advanced tumors include DNA-alkylating agents, anti-metabolites, anthracyclines, vinca alkaloids, topoisomerase inhibitors, and others and commonly target cycling cells with substantial side-effects in normal proliferative tissues like hair loss, diarrhea, and infections [5]. For certain types of tumors derived from hormonally responsive tissues, i.e., breast, endometrium, prostate, testes, thyroid, and adrenal cortex, hormone modulation and hormone receptor antagonists constitute effective anti-cancer therapy options [6].

With improved sequencing techniques and deeper molecular understanding of cancer pathogenesis, new targeted therapies could be developed-with the advantage of greater specificity towards molecular pathways altered in cancer cells compared with “classical chemotherapy” (recently reviewed in [7]). Apart from approved antibodies and kinase inhibitors, targeting either growth factor receptor tyrosine kinases (EGFR, PDGFR, VEGFR, c-MET et al.), downstream kinases (BRAF, MEK, AKT, mTOR et al.), or fusion proteins (BCR-ABL) dysregulated in tumors, a number of alternative small-molecule drugs and natural compounds are available for tumor therapy. Most recently, novel immunotherapies, in particular checkpoint inhibition with anti-CTLA-4 and anti-PD-1/-PD-1L antibodies targeting negative regulatory receptors on T cells, have been successfully applied against a growing number of tumor types [8]. Because intrinsic and acquired therapy resistance to approved cytotoxic and targeted therapies, and in part also to immunotherapies, still represent a major hurdle to long-term progression-free survival (PFS) and lasting remissions in cancer patients [7,8,9], different classes of small-molecule drugs are currently under investigation in pre-clinical and clinical trials in alternative combination regimens and will be discussed in this review.

## 2. Approved Targeted Therapy Regimens against Kinase Pathways and Their Limitations

In melanoma, the most common driver mutation occurring in over 50% of patients affects the serine/threonine protein kinase BRAF (BRAF^V600E^), a downstream effector of growth factor receptor signaling, that can be therapeutically targeted by specific BRAF^V600mut^ inhibitors (BRAFi) [10]. Because of the rapid development of acquired resistance during BRAFi monotherapy within months, combination therapy with BRAFi and MEK-inhibitors (MEKi) is currently standard in the treatment of BRAF^V600mut^-positive stage IV melanoma patients [11]. Combination treatment with BRAFi and MEKi has synergistic anti-tumor effects, reduced toxic side effects, and prolongs resistance development to a year on average. Depending on the presence of NRAS- or c-KIT-mutations, either MEKi, c-KIT inhibitors, immunotherapy with anti-PD-1 checkpoint blockers, or polychemotherapy are recommended for the treatment of advanced non-BRAF^V600mut^ melanoma [12]. Different mechanisms of acquired therapy resistance to either monotherapy with BRAFi or combination treatment could be identified in melanoma [8,13,14,15]: In response to BRAFi, reactivation of the mitogen activated protein kinase (MAPK) pathway, e.g., due to switching among BRAF, ARAF and CRAF isoforms, and enhanced insulin like growth factor (IGF)-IR/PI3K signaling occurred [13]. Reduced cytotoxicity mediated by upregulation of BCL-2 family pro-survival factors through activation of the PI3K/AKT pathway downstream of IGF-R or in context with activation of the hepatocyte growth factor (HGF)/c-MET or fibroblast growth factor receptor (FGF) pathway or via melanocyte master regulator microphthalmia associated transcription factor (MITF) was also observed under BRAFi and MEKi combination therapy. In addition, BRAFi and MEKi treatment may interfere with immune responses to melanoma cells.

Similarly, the targeted therapy combination with BRAFi and MEKi has recently been approved for the treatment of a specific type of metastatic non-small cell lung carcinoma (NSCLC) by the US Food and Drug Administration (FDA) [16]. In other types of lung cancer with epidermal growth factor receptor (EGFR) mutations, commonly tyrosine kinase inhibitors (TKi) against the mutant EGFR or anti-EFGR antibodies are applied alone or in new combination therapy trials with other anti-cancer therapeutics [17]. Combination therapy is also key to the treatment of metastatic colorectal cancer (CRC)-most frequently bearing KRAS, NRAS, BRAF, EGFR, and MYC mutations, loss of functions, amplifications, and microsatellite instability (MSI)-as reflected by ongoing clinical trials (e.g., HERACLES trial, NCT03225937) [18]. In general, mutations in key oncogenic kinase signaling pathways-including the RAS-RAF-MEK-ERK/MAPK, the PI3K/PTEN-AKT-mTOR, the JAK-STAT-or the WNT/β-catenin/MYC pathways occur with high frequency in a variety of tumors [2,3]. Because of parallel activation and significant signaling cross talk between these pathways, successive and combination therapies targeting different components with established small-molecule inhibitors based on an individual assessment of tumor or liquid biopsies may have the potential to overcome, or at least delay, therapy resistance (e.g., my pathway trial, NCT02091141 [7,19]). 

## 3. Alternative and Emerging Strategies for Improved Targeted Cancer Therapy

**Figure 1:**
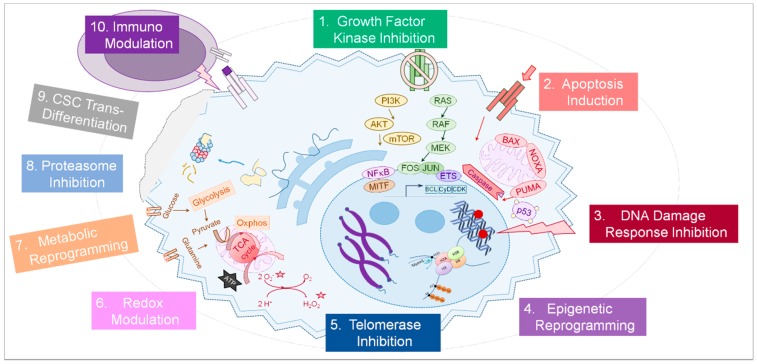
The principles of targeted cancer therapy. A number of physiological processes are potentially altered in a cancer cell (light blue) and can be targeted by small-molecules and other drugs (1–9, strongly simplified). In addition, immune cells (purple) can be targeted (10) as well as other cells of the tumor micro-environment (not shown). A more detailed description of each cellular process and the therapeutic ways to interfere with it is provided in the text.

Apart from oncogenic kinases in growth factor receptor signaling, a variety of cellular pathways are altered in tumor cells and may provide alternative druggable targets for tumor therapy (summarized in Figure 1). The following sections will provide an overview of molecular pathways and functions commonly altered in tumor cells in context with emerging strategies how to therapeutically interfere with these processes with small-molecule agents (Table 1). Moreover, selected current approaches targeting the tumor micro-environment—extracellular matrix, angiogenesis, or immune cells—will be addressed in brief [20]. With focus on treatment options for advanced melanoma and therapy resistance management, information about small-molecule tumor therapies and potentially beneficial therapy combinations in ongoing clinical trials was extracted from listings in public databases [19] and published research articles and will be highlighted.

### 3.1. Kinase Inhibitor Combinations

Based on a proven principle the use of new kinase inhibitors and inhibitor combinations may enhance established first line medications-because additional mutations or activation of compensatory growth factor signaling pathways are frequently observed in response to cancer therapy. To overcome the acquisition of melanoma therapy resistance commonly occurring on average after 12 months in patients on BRAFi and MEKi combinations [7], the ongoing LOGIC-2 clinical trial explores the effects of a third agent for the treatment of advanced BRAF^V600^-positive melanoma. Based on the genetic assessment of a tumor biopsy taken at progression of disease (PD) patients will receive either an inhibitor of cyclin-dependent kinases CDK4/6 (*ribociclib*, LEE011), of class I PI3K (*buparlisib*, BKM120), of the c-MET receptor tyrosine kinase (*capmatinib*, INC280), or of fibroblast growth factor receptor (FGFR) kinase (BGJ398) in a triple therapy approach (NCT02159066). Whereas the rationale for use of PI3Ki, METi, or FGFRi lies in the potential parallel activation of respective growth factor signaling pathways, adding CDKi may be beneficial for the treatment of tumors with elevated CDK4/6 activity or genetic alterations in the Cyclin D-CDK4/6-p16^INK4A^-RB pathway. In addition, a triple combination of BRAFi and MEKi with a heat shock protein 90 (HSP90) inhibitor (*onalespib*) is being tested in an ongoing phase I trial (NCT02097225). 

The efficacy of the combination of MEKi (*binimetinib*/MEK162) and CDK4/6 inhibitors is currently also assessed in patients with advanced KRAS-mutant non-small cell lung cancer (NSCLC) (*palbociclib*, NCT03170206)—and will be followed up on in NRAS-mutant melanoma based on promising phase Ib trial results (*ribociclib*, NCT01781572). In advanced squamous cell lung, pancreatic, head & neck and other solid tumors, combining the inhibition of CDK4/6 (*palbociclib*) and PI3K/mTOR (*gedatolisib*) may have advantage over the use of each drug alone or the Platinum-based chemotherapy (NCT03065062). For another type of NSCLC with activation of the c-MET pathway, the efficacy of MET inhibitors (*crizotinib* and *capmatinib*, INC280) is being assessed either as monotherapy (NCT02414139) or in combination with epidermal growth factor (EGF) tyrosine kinase inhibitor *erlotinib* (NCT01911507).

To target the signaling of angiogenic factors that control tumor neovascularization, including vascular epithelial growth factor (VEGF), PDGF, and FGF receptor tyrosine kinase signaling, oral multi-kinase inhibitors, such as *lucitanib*, have been tested in certain forms of NSCLC and breast cancer with genetic FGR alterations [21]. The combination of VEGF inhibitor *nintedanib* with *paclitaxel* chemotherapy may provide an alternative therapy regimen for patients with BRAF^WT^ metastatic melanoma (NIPAWILMA, NCT02308553). Similarly, inhibitors of focal adhesion kinase (FAK), a key regulator of integrin signaling, target tumor cell proliferation, invasion, metastasis, and angiogenesis and are promising drugs for combination therapy—in melanoma with BRAFi and in other tumors such as CRC with activated stroma—because of a limitation of tumor cell escape mechanisms [22,23]. Due to supporting effects of FAK inhibition on CD8^+^ T cell adaptive immune responses with potential synergy with anti-PD1 therapy in preclinical studies, a current trial explores the safety and tolerability of the combination of FAKi *defacitinib* with anti-PD1 antibody *pembrolizumab* in advanced solid tumors (NCT02758587).

### 3.2. Apoptosis Induction and Autophagy Modulation

Most anti-cancer therapies directly or indirectly exploit “programmed cell death” (apoptosis) and other cell death pathways. However, during step-wise transformation, tumor cells acquire various genetic alterations to reduce their sensitivity to cell death and increase their survival under stress conditions—limiting the efficacy of “cell death drugs” at doses that will not harm healthy cells [1,2,3]. A number of emerging therapeutics therefore focus on “reactivating” cell death programs in tumor cells. In general, apoptosis, the so called “programmed cell death”, can be initiated by an extrinsic (death receptor) and an intrinsic (mitochondrial) death pathway—both leading to a common death executing program mediated by the Caspase family of proteases (reviewed in [24]). Accordingly, drugs that either trigger extrinsic death receptor signaling or enhance intrinsic mitochondrial pathways are currently tested for clinical use in tumor therapy. In addition, inhibition of autophagy may enhance tumor cell apoptosis.

Because death receptors (DR), including CD95 (Fas/APO1), DR3, DR6, TNF-R1, and TNF-related apoptosis-inducing factor (TRAIL)-R1/DR4 and TRAIL-R2/DR5, depending on their expression, have the ability to trigger apoptosis in most tumor cells, strategies to activate death signaling via DR agonists or agonistic antibodies have strong therapeutic potential against cancer. In melanoma, due to variable expression of TRAIL-R1/DR4, TRAIL-R2/DR5, and other DRs, the choice and specificity of the agonistic antibody as well as its ability to crosslink Fcγ receptors on myeloid cells turned out to be crucial for effective induction of apoptosis signaling and therapeutic efficacy [25]. An ongoing clinical study investigates the potential of a TRAIL/DR5 antibody (*DS-8273a*) to augment the clinical efficacy of PD1-blocker *nivolumab* in melanoma combination therapy (NCT02983006).

Different classes of small-molecule drugs enforce the intrinsic mitochondrial pathway: On the one hand, by mimicking the natural antagonists of BCL-2 family survival proteins (so called BH3-mimetics or BCL-2 family inhibitors) or of the inhibitors of apoptosis (IAPs) (so called SMAC-mimetics); on the other hand, by directly targeting BCL-2 expression with antisense oligonucleotides—all sensitizing tumor cells to death [26]. In melanoma and other tumors, resistance to kinase inhibitors like BRAFi and MEKi is often mediated by an abrogation of intrinsic apoptosis signaling pathways—namely by downregulation of BH3-only proteins or by induction and activation of BCL-2 family members like BCL-2, BCL-X_L_, BFL-1, and MCL-1 [27]. While melanoma and other solid tumor are mostly insensitive against mono-therapy with either BH3-or SMAC-mimetics, combination approaches of kinase inhibitors with apoptosis mimetics or epigenetic drugs inducing BH3-proteins (see Section 3.4) have the potential to override drug resistance. In melanoma resistant to BRAFi and MEKi, in particular BH3-mimetics targeting MCL-1 and manipulation of the MCL-1/NOXA-axis maybe beneficial and warrant clinical testing [28]. The BH3-mimetic *venetoclax*, selectively inhibiting BCL-2, often overexpressed in leukemia and lymphoma, has been approved for the treatment of refractory chronic lymphatic leukemia (CLL) [29]. Currently, this drug is being tested in combination with *navitoclax* and chemotherapy in acute lymphoid leukemia (ALL) (NCT03181126) and with various other targeted therapeutics in different types hematologic malignancies. In addition, a first Phase-Ib dose-finding study evaluates the SMAC-mimetic *debio 1143* in combination with anti-PD-L1 antibody avelumab in patients with advanced solid malignancies, especially NSCLC after Platinum-based therapy (NCT03270176).

Another promising strategy to enhance tumor cell apoptosis involves the “re-activation” of tumor suppressor p53, a key player in apoptosis and cell cycle arrest that is frequently mutated and inactivated in various cancers [30]. Restoration of p53 function in melanoma, often expressing inactivated wildtype p53, and in other tumors can be archived by targeting its antagonists, E3 ligase Mdm2, Mdm4, Mdmx, and inhibitors of apoptosis stimulating protein of p53 (iASPPs) [31] or through direct p53 activators [32]. p53 reactivation may have synergistic effects with BRAFi potentially overcoming therapeutic resistances. In a current phase-I dose-escalation study, an MDM2 antagonist (RO6839921) is intravenously applied in patients with advanced malignancies, including acute myeloid leukemia (AML) (NCT02098967).

In addition, targeting autophagy, another homeostatic cellular process pathway involved in survival and proliferation of tumor cells, with established drugs, such as the anti-malaria agent *hydroxychloroquine* (HCQ), may induce tumor cell apoptosis and enhance the potency of many cancer therapies [33]. Hence, several ongoing clinical trials are conducted to evaluate the anti-tumor effects of autophagy inhibition in a variety of solid tumors in mono- or combination therapy. These include clinical studies assessing the safety and efficacy of the combination of *hydroxychloroquine* with histone deacetylase (HDAC) inhibitor *vorinostat* in refractory metastatic colorectal cancer (mCRC) (NCT02316340) and in other advanced solid tumors (NCT01023737, also see Section 3.4).

### 3.3. DNA Damage Response Inhibitors

In response to DNA insults caused by endogenous and exogenous stressors—including reactive oxygen species (ROS), stalled replication (RS), DNA-damaging drugs, or irradiation—normal cells and tumor cells activate so called DNA-damage response (DDR) pathways. Among the principle outcomes of DDRs are either transient cell cycle arrest and restoration of the damaged nucleotide-sequence of the DNA (DNA repair) or apoptosis and clearance of the damaged cells (reviewed in [34]). In a variety of tumors, functional inactivation of such DDR pathways contributes to increased genomic instability and mutational load, but also sensitizes cells towards standard genotoxic (DNA damaging) treatments—providing a rationale to explore the use of DDR inhibitors in novel combination therapy regimens.

Among the main therapeutic targets for DDR inhibitors, currently being tested in phase 0‒II clinical trials, are the phosphatidyilinositol-3-OH-kinase (PI3K) family kinases ataxia teleangiectasia mutated (ATM) and RAD3-related (ATR) and their downstream DDR kinases CHK1 and WEE1 [35]. In addition, inhibitors of poly(ADP-ribose) polymerase (PARP), an enzyme involved in base excision repair (BER) of single strand breaks (SSB), are clinically evaluated singly or in combination with chemotherapy [36]. In brief, ATM kinase is activated in response to double strand breaks (DBS), initially recognized by the so called MRN-complex (composed of MRE11, RAD50, and NBS1). ATM then phosphorylates histone H2AX on serine residue S139 close to the break (γH2AX) as an amplifier to recruit more MRN-molecules and additional DNA repair proteins like BRCA1 and 53BP1. This leads to the activation of the G1-S cell cycle checkpoint and p53-dependent induction of CDK-inhibitor p21 ^WAF1/CIP1^ as well as apoptosis genes. In contrast, single stranded DNA (ssDNA), arising at either stalled replication forks or at resected DSB, causes activation of ATR kinase that mediates cell cycle arrest and DNA repair at the break via downstream kinase CHK1. In addition, PARP acts as “molecular sensor” to identify SSB and contributes DNA repair.

Despite some limitations in the use of DDR inhibitors due to lipophilicity and toxicity in proliferating normal cells, the concept of synthetic lethality based on mutations in DDR pathways in tumor cells can be exploited therapeutically. Based on the enhanced sensitivity of tumor cells with inactivated tumor suppressor genes BRCA 1 and 2 towards PARP-1 inhibition [37], the PARP inhibitors *olaparib*, *rucaparib*, and *niraparib* could recently receive FDA approval for the treatment of BRCA-mutant ovarian and refractory breast cancers. Similarly, due to mutations in the p53/RB-pathway, a deregulated G1 cell cycle checkpoint, and increased replication stress tumor cells often fully depend on the S/G2 checkpoint. This generates a therapeutic window for ATR and CHK1 inhibitors and increases the sensitivity to DNA damaging drugs [38]. ATM inhibitors have been shown to be effective in pre-clinical studies in brain and prostate cancers in combination with chemo-or radiotherapy with greater sensitivity of p53-mutant or PTEN-mutant cells, respectively [39]. Following up on the pre-clinical synthetic lethality of ATM loss-of-function in combination with drugs inhibiting either growth factor kinases MEK1/2 or PARP [40,41,42], a current phase-I clinical trial assesses the ATM inhibitor *AZD01566* alone or in combination with PARPi *olaparib* or chemotherapy in patients with advanced solid tumors (AToM, NCT02588105). Similarly, the combination of CHK1 inhibitor prexasertib and PARP inhibitor *olaparib* in advanced solid tumors is tested based on their synergistic effect against cancer cells in vitro (NCT03057145). In melanoma, pre-clinical studies suggest that inhibition of PARP and DNA damage responses is a promising strategy for combination therapy [43]. In summary, enhanced effects of DDR inhibitors in cells with sensitizing mutations and synergistic effects of combinations of different checkpoint inhibitors warrant further clinical testing.

### 3.4. Epigenetic Drugs

Cellular transformation and tumor cell plasticity require specific epigenetic changes occurring in response to environmental and intrinsic stimuli. Often, epigenetic modifications in tumor cells are involved in the silencing of tumor suppressor genes—at different stages of cellular dedifferentiation, during epithelial-mesenchymal transition (EMT), and during drug resistance development (reviewed in [44]). Therefore, DNA-methylation patterns, histone modifications and altered microRNA expression not only represent valuable biomarkers, but in particular, DNA methylation via DNA methyltransferases (DNMTs) and histone deacetylation via HDACs have also been explored as therapeutic targets for combination therapy. More recently, additional histone modifying enzymes and histone code readers of the bromodomain-extraterminal (BET) family have come to the spotlight as potential weapons against tumor resistance to kinase inhibitors.

DNA hypomethylating agents such as 5-azacytidine and 5-aza-2-deoxy-cytidine (*decitabine*), both inhibitors of DNMT1, have been clinically approved for the treatment of hematological malignancies [45] and induce differentiation und apoptosis due to reactivation of cell cycle regulating genes. In BRAF-mutated metastatic melanoma, a phase I–II clinical study assess the effect of DNA methylation on tumor progression and resistance development under therapy with BRAFi and MEKi with or without *decitabine* (NCT01876641). Similarly, the drug may induce cancer stem cell differentiation and thereby aid in combination regimens (see Section 3.9).

Histone deacetylase inhibitors (HDACi) were also among the first anti-cancer drugs targeting epigenetic enzymes that received approval for the treatment of hematological malignancies. The first HDACi, *vorinostat*, approved as third line therapy for cutaneous T-cell lymphoma (CTL), is currently clinically tested as adjuvant treatment of CRC in combination with *hydroxychloroquine* (see Section 3.3) against TKi *regorafenib* (NCT02316340) and in advanced solid tumors (NCT01023737). In addition, the combination of *vorinostat* with immunotherapy using checkpoint inhibitor *pembrolizumab* is tested in patients with advanced NSCLC (NCT02638090). Other HDACi, including *belinostat*, *panobinostat*, and *etinostat*, are currently evaluated for the treatment of refractory solid malignancies, including mesothelioma, melanoma, lymphoma, and others–in part in combinations with other targeted drugs or with classical chemotherapy [20,46]. In a subset of drug-resistant melanoma, HDACi may restore BRAFi sensitivity via different mechanisms, including induction of pro-apoptotic pathways and decreased PI3K signaling [47]. In addition, histone-independent actions of HDACi, in particular the hyperacetylation of tumor suppressor p53, chaperone HSP90, and NF-κB subunit p65/RelA, may contribute to their effects on drug-resistant tumor cells and reflect the broad impact of HDAC/i on cancer in general [48]. Consequently, safety and efficacy of HDACi will currently be evaluated in particular in “problem” melanoma and tumors, such as resistant BRAF^V600mut^ advanced melanoma (*vorinostat*, NCT02836548), high risk uveal melanoma (NCT03022565), and in combination therapies with checkpoint inhibitor immunotherapy (PEMDAC, NCT02697630, NCT02032810 and NCT02437136)—seeking to overcome therapy resistance.

Closer analyses of the critical roles of different histone modifying enzymes and histone readers in cancer progression and therapy resistance have triggered the development of additional inhibitors. Drugs targeting the polycomb repressive complex (PRC) 2 factor EZH2, catalyzing H3K27 trimethylation (EZH2i), or BET protein family epigenetic readers, affecting transcription of genes with super-enhancers (BETi), are already being tested in clinical studies in different malignancies [49]. Due to their potential to induce apoptosis in melanoma cells by increasing NOXA and AIF and decreasing MCL-1 levels, EZH2 inhibitors may enhance the sensitivity to MAPK inhibition in combination regimens with BRAFi and MEKi. In current early clinical trials, the EZH2 inhibitor *tazemetostat* is assessed in relapsed or refractory malignant mesothelioma with BAP1 loss of function in adults (NCT02860286). However, due to a case of secondary lymphoma, new enrollment of pediatric patients in current *tazemetostat* trials for synovial sarcoma (NCT02601937) or different relapsed or refractory advanced solid tumors with sensitizing mutations is currently suspended (NCT03213665). In principle, BET inhibitors (BETi) as well may override resistance to MAPK inhibitors in certain tumors (such as NRAS-mutant melanoma) by sensitizing them to apoptosis and have been shown to potently inhibit growth of solid and hematological tumor cells in vitro and in vivo in animal models [50]. According to dose findings studies, BETi *OTX015* can be safely applied in patients with acute leukemia (NCT02698189) or selected advanced solid tumors including triple negative breast cancer (TNBR), NSCLC, and castration-resistance prostate cancer (CRPC) and pancreatic cancer (NCT02698189). In additional ongoing phase I dose-escalation studies, other BETi are tested in advanced or recurrent solid malignancies (NCT02630251, and NCT02419417) and in hematologic malignancies (NCT02543879), as well as in a phase I/IIa study as monotherapy or in combination with *nivolumab* immunotherapy (NCT02419417). In other regimes, combinations of BETi with inhibitors of cyclin dependent kinases (CDKi) or HDACi or ATMi or PARPi maybe tested -following up on promising synergistic anti-tumor effects in vitro. Moreover, in pre-clinical studies novel roles of enzymes, either affecting the ubiquitination of histone H2A K119, such as PCR1 factor BMI1, 2A-DUB/MYSM1, and RNF2 or the methylation of H3 K9, in progression and drug resistance of melanoma and other cancers are currently being investigated [51,52,53].

On the other hand, clinical testing of the safety of microRNA MRX3 in a dose-escalation study in patients with unresectable primary liver cancer, advanced or metastatic cancers, or hematological malignancies was recently terminated due to several serious immune related events (NCT01829971). Current clinical studies mainly focus on microRNAs as biomarkers [20].

### 3.5. Telomerase Inhibitors

Immortalization of tumor cells usually requires the induction and activation of telomerase to maintain telomere length and integrity and to prevent DNA damage responses [54]. Consequently, genes involved in telomere maintenance—including telomerase reverse transcriptase (hTERT, the catalytic subunit of telomerase), shelterin complex proteins (TRF1, TRF2, POT1, TIN2, TPP1, and Rap1), and telomerase-associated proteins (such as hTEP, p23, HSP90 and dyskerin)—are relatively frequently activated or mutated in tumors including both familial and acquired melanoma, often in context with BRAF mutations (recently reviewed in [55,56]).

Therapeutic inhibition of telomerase activity can be achieved on the one hand by targeting either the hTERT catalytic subunit—by nucleoside analoga such as 3′-azido-2′,3′-dideoxythymine (AZT)—or on the other hand, the telomerase RNA (hTR) component—via antisense oligonucleotides or the more specific telomerase template antagonists such as the 13-mer oligonucleotide N3′-P5′-thio-phosphoramidate (GRN163L, *imetelstat*) [55,57]. Telomerase enzyme inhibition and targeting of hTR in cancer cells generally result in progressive telomere shortening and reduced cellular viability. However, despite significant inhibition of tumor growth and metastasis, in line with off-target effects of telomerase inhibitors (Ti) in in vitro studies [58], a number of clinical trials had to be withdrawn due to unfavorable toxicity profiles. For instance, *imetelstat* inhibited primary tumor growth and metastases in in vitro and in vivo studies. But in early phase clinical studies in adults and children, dose-limiting higher grade hematologic cytotoxicity and cases of intratumoral hemorrhage secondary to thrombocytopenia were the causes of premature cancellation [59,60]. An alternative approach to target telomeres in cancer cells is the use of so called “T-oligos”—guanine-rich oligonucleotides homologous to the 3′ telomere overhang sequence. T-oligos, in particular a specific 11-base oligonucleotide (5′-dGTTAGGGTTAG-3′ or T11), have been shown to induce DNA damage responses (DDRs) such as senescence, apoptosis, and cell cycle arrest in numerous cancer cell types, including melanoma, with only minimal cytostatic effects in normal cells [61]. Clinical testing of T-oligos may therefore be warranted. In addition, peptides derived from TERT have been in focus in anti-cancer immunotherapy—either to raise CD8^+^ cytotoxic T cell responses against TERT-epitopes on tumor cells [62] or to monitor tumor-specific anti-telomerase-specific CD4^+^ T cell immunity to TERT-neoantigens before and after chemo-or immunotherapy (Telocap02, NCT02846103).

### 3.6. Redox Modulators

By definition reactive oxygen species (ROS) occurring as byproducts of cellular metabolism or due to exposure to external stressors are oxygen species with reactive properties (such as peroxides, superoxide, hydroxyl radicals and singlet oxygen)—that can damage other cellular molecules including proteins and DNA if insufficiently detoxified by cellular antioxidant systems. In tumor cells, in brief, ROS are “double-edged swords”: on the one hand, ROS have tumor-suppressive functions due to the induction of tumor cell apoptosis. On the other hand, ROS may promote tumorigenesis through induction of DNA damage and mutations, as well as epithelial-mesenchymal transition (EMT) and metastasis by regulating extracellular matrix and cytoskeleton remodeling [63]. Because oxidative stress responses interfere with a complex network of cellular mitochondrial detox enzymes, transcriptional regulators, and tumor suppressors—including NFκB, nuclear factor erythroid 2 like 2 (NRF2), and BRCA1—therapeutic strategies targeting with these processes with small-molecules or neutraceuticals and phytochemicals may hold promise for targeted tumor combination therapy and may reduce cytotoxicity of conventional therapies.

The redox modulator *dimethyl fumarate* (DMF), an approved treatment for autoimmune diseases such as multiple sclerosis and psoriasis, induces recycling of intracellular antioxidant glutathione (GSH) pools, inhibits NFκB p65/RelA and activates NRF2—thereby overall promoting anti-oxidant responses in different cell types [64]. Current clinical trials therefore assess the safety and suitable dose of DMF to reduce cytotoxicity in combination with radiotherapy (RT) and temozolomide (TMZ) in glioblastoma multiforme (GBM) (NCT02337426) and in other cancers [65]. In addition, DMF has been shown to induce apoptosis in T cell lymphoma cells [66] and is currently being tested in phase-I-clinical trials in refractory chronic lymphocytic leukemia (CLL) (NCT02784834). In melanoma, anti-proliferative and pro-apoptotic effects of DMF could be demonstrated leading to reduced growth and metastasis of melanoma in pre-clinical models [67,68]. Similarly, the selective GSK-3β inhibitor *thiadiazolidinone* (TDZD-8) has been shown to selectively cause death of stem cell marker expressing leukaemia cells through depletion of thiols with rapid accumulation of ROS—with only minimal toxicity to normal hematopoietic cells [69]. TDZD-8 may undergo clinical testing.

An example for the use of vitamins with antioxidant properties as alternative anticancer therapies is a phase II-clinical trial assessing the efficacy of high dose vitamin C (*sodium ascorbate*) infusions to synergistically act with chemotherapy to kill cancer cells and to reduce toxic side effects (NCT02655913) [70]. In addition, other vitamins and antioxidants, such as vitamin E, *N*-acetylcysteine (NAC), green tee catechins, and others, are being assessed in combination regimens. Because bioenergetics and cellular metabolism are connected, the drugs discussed in the next section also in part interfere with the redox modulation and energy requirements of tumor cells.

### 3.7. Metabolic Reprogramming Drugs and Enzymatic Inhibitors

To maintain high proliferation rates under nutrient- and oxygen-limited conditions, tumor cells need to adapt their nutrient transport, metabolism, and bioenergetics—a process known as “metabolic reprogramming” in cancer biology. On the one hand, oncogenic signaling pathways, such as the MAPK pathway, regulate the use of glucose and amino acids and the switch from oxidative phosphorylation (oxphos) to anaerobic glycolysis (known as “Warburg effect”) in tumor cells (reviewed in [71]). On the other hand, nutrient metabolism interferes with responsiveness to BRAF (kinase) inhibition in melanoma and other tumors [72]. Induction of anabolic pathways supporting tumorigenesis often results from sustained activation of the PI3K/Akt/mTOR pathway, mTOR complex 1 (mTORC1), and transcriptional networks involving HIF-1α/MYC/SREBP1. Therefore, strategies interfering with both, glucose and amino acid supplies, and the regulating anabolic signaling pathways, may be therapeutically effective against tumors in a relatively selective manner and are currently explored in pre-clinical and clinical settings.

One approach to specifically interfere with cancer cell glucose and energy metabolisms involves the application of the anti-diabetic biguanides *metformin* and *phenformin* that exhibit anti-cancer properties by interfering with mTOR signaling and inhibiting mitochondrial complex I [73]. In the presence of biguanides, tricarbocylic acid (TCA) cycle intermediates and mitochondrial ATP production are reduced leading to tumor cell death when glycolytic ATP levels decrease as a result of limited glucose availability to tumor cells. In melanoma cell cultures, *phenformin* reduced cellular viability and growth of both CSC and non-CSC and abrogated invasion more potently than *metformin* [74]. Current phase-I clinical trials therefore assess the safety and dose-escalation options for the use of *phenformin* in combination with BRAFi and MEKi therapy in metastatic melanoma (NCT03026517), as well the combination of *metformin* and *temsirolimus*, an mTOR inhibitor, in advanced cancers (NCT01529593). Another metabolic drug with anti-cancer activity is the pyruvate mimetic compound *dichloroacetate* (DCA), an established treatment for pediatric mitochondrial disorders. DCA stimulates mitochondrial function by inhibiting regulatory pyruvate dehydrogenase kinases (PDK) 1–4 at the expense of glycolysis to reverse the Warburg effect and block the growth advantage of tumor cells [75]. Ongoing clinical studies evaluate the effects of DCA vs. placebo in combination with *cisplatin* and radiation treatment in patients with stage III–IV squamous cell carcinoma of the head and neck (SCCHN) (NCT01386632). In pre-clinical studies, inhibition of glutamine transport and uptake were suggested as potential therapeutic strategies in multiple myeloma [76] and both, BRAF^WT^ and BRAF^V600mut^, melanoma. Based on similar principles, the benefits of nutraceuticals such as vitamins, curcumin, and green tee polyphenols—interfering with metabolic and redox balances—are clinically explored in treatment and prevention of various cancers [77].

### 3.8. Proteasome Inhibitors (PI)

The ubiquitin-proteasome pathway, regulating the proteolytic degradation of proteins involved in cell cycle control und survival (including cyclins, p53, NF-κB, and others), is involved in many aspects of tumor cell transformation and tumor growth and also has important roles in normal cells (reviewed in [78]). Toxicities and acquired resistances have therefore so far limited the use of this drug class—with a few exceptions. Clinical evaluation of the first-generation reversible small molecular (26S) PI *bortezomib*, already approved as second line treatment for multiple myeloma and for mantel cell lymphoma [79], may hold promise for therapeutic use in combination with HDAC inhibitor *vorinostat* or chemotherapy in NSCLC or with purine nucleoside metabolic inhibitor clofarabine in other refractory solid tumors (NCT02211755). However, in metastatic melanoma, the combination of *bortezomib* with *paclitaxel* and carboplatin was associated with considerable cytotoxicity and had only limited clinical benefit [80]. Newer second-generation PIs with improved selectivity and novel combinations restoring apoptotic pathways may have better clinical efficacy.

### 3.9. Cancer Stem Cell Transdifferentiation

Tumor heterogeneity and cellular plasticity towards environmental changes confer critical survival advantages to a tumor cell population and limit lasting therapeutic success [81]. In particular, the slow-cycling population of cancer stem cells (CSC), also called “cancer-initiating” cells, has been implicated in driving relapses during targeted and conventional cytotoxic tumor therapy [82]. CSC may therefore constitute important targets for combination therapies or two-component drugs targeting both proliferating and SC tumor cells. Exploiting their stem cell properties, the so called “transdifferentiation” therapy aims at inducing terminal differentiation of CSC towards benign cells of the original lineage the tumor arose from or to related lineages through pharmacological manipulation of transcription factor balances.

One such differentiation strategy involves the use of retinoids: the retinoid acid receptor (RAR) agonist all trans-retinoid acid (*ATRA*) has been successfully applied in acute promyelocytic leukemia (APL) [83]. Moreover, the selective retinoid X receptor (R(X)R) agonist *bexarotene* may constitute a weapon against multidrug-resistance in breast cancer [84]. Similarly, retinoids may induce differentiation in other cancer stem cell types. In melanoma, the balance of master regulator microphthalmia-associated transcription factor (MITF) and tyrosine kinase AXL has been shown to function as rheostat determining the transition between proliferative and invasive state—with highest MITF levels promoting neuronal differentiation [85]. In addition, either high MIFT-levels or a low MIFT/AXL-ratio were shown to be associated with resistance of BRAF-and NRAS-mutant melanoma cells towards kinase inhibitors and other targeted therapies [86]. Because *ATRA* treatment of melanoma cells in culture induced differentiation and apoptosis in context with increased expression of MITF [87], *ATRA* may provide a combinational therapeutic tool against certain melanomas—to tackle both, melanoma SC and therapy resistance, via key regulator MITF and related gene expression. As a single agent, on the opposite, R(X)R agonist *bexarotene* was not efficient against metastatic melanoma in an earlier trial [88]. Biomarkers as well as effects on immune cells may have to be considered in future anti-cancer therapy approaches using retinoids.

Second, certain epigenetic drugs (see Section 3.4) have the potential to regulate tumor cell transdifferentation, in particular DNA demethylating agents and HDACi [89]. In this respect, the DNA hypomethylating drug 5-aza-(2-deoxy-)cytidine (*decitabine*) induced the neuronal marker gene microtubule associated protein 2 (MAP2), progressively methylated during melanoma progression, in metastatic melanoma cells. However, in a phase II clinical trial 5,6-dihydro-5-azacytidine (DHAC) demonstrated only limited benefit against malignant melanoma—without serious myelosuppression. Contrarily, decitabine plus high dose interleukin-2 induced regression of melanoma in 31% of patients, but was associated with significant occurrence of neutropenia. In addition, decitabine is tested in combination with BRAFi and MEKi to measure the time to progression under this combination (NCT01876641). Similarly, HDACi have been shown to promote MAP2 expression and induce benign neuron-like differentiation in a metastatic melanoma mouse cell line in vitro [90]. HDAC inhibitors also inhibit the growth of uveal melanoma cells both in vitro and in vivo, and other problem melanoma types (see Section 3.4).

A promising alternative transdifferentiation strategy is to induce adipocyte-like differentiation of tumor cells by application of unsaturated fatty acids—such as oleic, palmitoleic, or linoleic acid—or peroxisome proliferation-activated receptor gamma (PPARγ) agonists. This approach has successfully been used to enforce transdifferentiation in melanoma spheres and in many tumor cell lines in vitro [91] and will now need to be transferred to clinical applications. Although a growing number of in vitro studies could demonstrate significant effects, currently, there is no clinically approved fixed transdifferentiation therapy regimen for cancers yet.

### 3.10. Immunomodulatory Drugs

Apart from the inhibitory surface receptors CTLA-4 and PD-1 several cytoplasmic proteins, such as ubiquitin ligases and kinases, downstream of the T cell receptor (TCR) act as negative regulators of T cell activation and may limit anti-tumor immune responses. Cytoplasmic inhibitors of TCR signaling include Casitas B cell lymphoma (CBL) family E3 ligases (c-CBL, CBL-b, ITCH, GRAIL, DELTEX, and NEDD), adaptor proteins DOK-1/2 and STS1/2, as well as kinases such as DRAK2, and others (reviewed in [92]). Using siRNA and shRNA approaches to down-modulate *CBL*-*b* in T cells, increased T cell activity towards melanoma cells could be achieved in pre-clinical models [93]. Although no clinically approved inhibitors are currently available, targeting intracellular negative regulators of TCR signaling may represent a promising alternative anti-tumor strategy for combination immunotherapy as it simultaneously deactivates several adaptive immune “checkpoints” including TFG-β, CTLA-4, and PD-1 signaling [94,95]. According to pre-clinical studies, avidity-tuning through manipulation of negative TCR regulation may also enhance target cell selectivity in adoptive chimeric antigen receptor (CAR) T cell cancer therapy [96]. Interestingly, targeting CBL E3 ligases may also have synergistic effects in certain tumor cell types, such as melanoma, where c-CBL has recently been shown to promote tumor growth and mobility in part via the focal adhesion kinase (FAK)-GRB2-SRC nexus. Overall, immuno-stimulatory and growth-inhibitory effects therefore provide a rationale to further explore the therapeutic value of inhibiting these ubiquitination proteins in combination therapy of melanoma and other tumors [97].

An alternative strategy to limit negative regulation of T cell function and to break immunotolerance to tumor cells involves the modulation of L-tryptophan metabolism via the kynurenine pathway with the indoleamine 2,3-dioxygenase-1 (IDO1) inhibitor *epacadostat* [98]. However, despite initial excitement, the phase III-clinical trial of *epacadostat* with *pembrolizumab* for melanoma was recently halted because the combination therapy missed the first primary endpoint of improving PFS vs. *pembrolizumab* alone (ECHO-301/KEYNOTE-252, NCT02752074). Another ongoing clinical trial assesses the benefit of *epacadostat* administration in context of a multipeptide melanoma vaccine (NCT01961115). Moreover, HSP90 inhibitor therapy may work as indirect “immune adjuvant” leading to increased therapeutic T cell recruitment against EphA2 expressing tumors, such as BRAFi-resistant melanoma, due to transient proteasome-dependent degradation of HSP90 client protein EphA2 in absence of the functional chaperone [99,100].

### 3.11. Other Substances

A few other small-molecule anti-tumor therapeutics are currently being tested clinically and will not be discussed in more detail here. In brief, dose-finding studies are ongoing for WNT/β-catenin inhibitors (LGK974) as single agent or in combination with anti-PD1 antibody PDR001 (NCT01351103). In melanoma, altered expression and functions of WNT pathway genes including Frizzled receptors and their functions are ongoing areas of research. To follow up on an alternative strategy to destabilize survival proteins in cancer cells by interfering with cellular chaperones, a number of HSP90 inhibitors are tested in combination with BRAFi and MEKi in melanoma (NCT02721459), in NSCLC (NCT01784640), and in other cancers [101].

## 4. Towards Algorithms for Improved Combination Therapy and Individualized Approaches

In the light of the different targeted therapy strategies against cancers available, new possibilities arise to (1) overcome therapy resistance to current kinase inhibitor combinations by adding a third or fourth agent, (2) treat tumors not bearing the most common driver mutations (such as BRAF^WT^ melanoma) and “problem” tumors (such as mucosal or uveal melanoma or brain metastases), (3) find alternative strategies for individual patient needs (such as elderly patients, children, or patients with immune or metabolic disorders), e.g., by reducing cytotoxicity to normal cells. (4) to enhance established immuno-and chemotherapy regimens.

**Figure 2:**
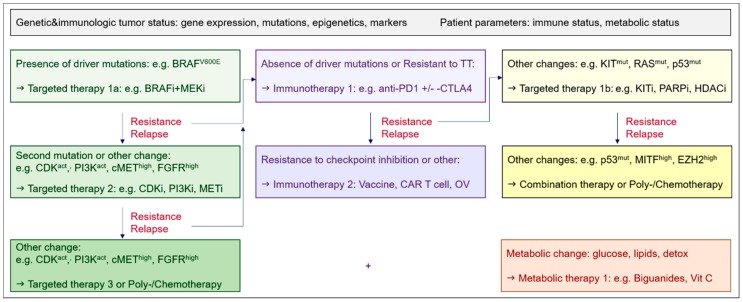
Algorithm suggestion for therapy decisions based on the assessment of the tumor genetic and immunologic status and patient parameters for the example of metastatic melanoma.

According to ongoing clinical trials, in BRAF^V600mut^ melanoma, therapy resistance could be addressed by either using triple combinations of kinase inhibitors (see Section 3.1), or combinations of BRAFi and MEKi with either apoptosis inducing drugs (death receptor agonists, BH3-or SMAC-mimetics, see Section 3.2.) or epigenetic drugs (DNMT1i, HDACi, EZH2i, BETi, see Section 3.4)—promoting tumor cell killing—or with metabolic drugs (*phenformin*, Section 3.7). Commonly, acquired resistance to kinase inhibitors may involve compensatory activation of other growth factor signaling pathways or additional kinase mutations, increased survival signaling, or altered cell cycle control [9]. To interfere with increased BCL-2 or MCL-1 in BRAFi-resistant melanoma and other cancer types, alternatively, different BH3-mimetics or epigenetic drugs may be applied—the later with potential additional benefit of impacting cell cycle regulation, EMT, and CSC transdifferentiation. To determine the most favorable individualized therapy regimens therefore, the current trend is towards a genetic assessment of tumor biopsies or “liquid biopsies” [102]. Based on patient parameters and molecular characterization of tumor biopsies, potentially from different metastases, or even single-cells [103], the design of individualized regimens will be optimized—as exemplified for the therapy of advanced melanoma (Figure 2).

In addition, alternative agents, such as DNA damage response inhibitors (PARPi, ATMi), newer telomer-based drugs (“T-oligos”), or anti-angiogenic strategies (VEGFi) could be applicable for combination therapies of BRAF^WT^ melanoma. These drug classes might also be promising for combination therapies with kinase inhibitors in other advanced cancers. Importantly, several of the emerging small-molecule approaches reviewed here are not directed against tumor-specific mutant proteins, but instead rather target metabolic, gene expression, or epigenetic changes more commonly occurring across different tumor types.

Although increased toxicity of targeted therapy combinations is a concern, molecular analyses of tumor biopsies may identify aberrations rendering tumor cells particularly sensitive to a specific drug combination as well as guide the use of combinations with non-overlapping toxicity profiles. Accordingly, preliminary results of clinical studies of novel kinase inhibitor combinations containing CDKi suggest that presence of alterations in the Cyclin D-CDK4/6-p16^INK4A^-RB axis confers increased tumor cell responsiveness—which may lead to overall manageable safety profiles of these combinations and favorable efficacies [104]. Similarly, adding retinoids or certain epigenetic drugs in combination regimens may have synergistic anti-tumor effects without increasing toxicity for the patient because of non-overlapping side effects [105]. The awaited results of ongoing clinical trials assessing the safety and tolerability of novel triple combinations of small molecule drugs or of combinations with conventional or immune therapies will therefore guide future therapy decisions. Moreover, therapy strategies that were previously not successful against a broader number of cancer types as monotherapy due to toxic side effects (such as proteasome or telomerase inhibitors)—might be applicable at lower doses in combination regimens or in modified forms.

Importantly, newer small-molecule targeted therapies may also help to enhance immunogenicity of tumors for immunotherapies or to reduce side effects of established chemo- and radiotherapy regimens. Because the combination of immunotherapy using checkpoint inhibitors with kinase inhibitors such as BRAFi for metastatic BRAF^V600mut^ melanoma may be effective, clinical trials assessing the best sequence, dose, and duration of treatments to archive the highest response rates are currently underway (NCT02818023, NCT02130466) [106].

## 5. Conclusions and Outlook

A variety of small-molecule targeted therapies are currently available for novel combinational and alternative therapy regimens based on individual patient and tumor parameters. Therefore, it is now important to establish additional biomarkers including gene mutation patterns, expression changes, epigenetic makers, liquid markers, and tumor micro-environmental markers—to improve the prediction of the best combination regimen for each patient, tumor, and therapeutic stage. In the treatment of advanced melanoma, both additional triple combinations with BRAFi and MEKi and successive application of targeted therapies, as well as combinations of small-molecules with immunotherapy, chemotherapy, and radiotherapy may open novel perspectives. Furthermore, identification of additional tumor biomarkers may also lead to improved prevention and adjuvant strategies.

## Figures and Tables

**Table 1:** Targeted tumor therapy strategies, selected agents, and their molecular targets in selected ongoing clinical trials.

Principle/Substance Group	Molecular Target	Cancer Type/Ongoing Trials (Selection)
1. Signaling/Kinase inhibitors		
- RAF-MEK-MAPK pathway inhibitors	BRAF^V600mut^, MEK	MM (LOGIC-2), NSCLC, CRC
- PI3K pathway inhibitors	PI3K, AKT, mTOR, GSK3	MM (LOGIC-2), advanced solid tumors
- Cell cycle kinase inhibitors	Cyclin-dependent kinases (CDK4/6)	MM (LOGIC-2), NSCLC, solid tumors
- Growth factor signaling inhibitors	Oncogenic receptor kinases	Advanced cancers (multikinase inhibitors)
	- FGFR	MM (LOGIC-2), TNBC, NSCLC
	- MET kinase	MM (LOGIC-2), RC, NSCLC, lymphoma
	- EGFR	NSCLC, CRC
	- VEGFR, PDGFR, others	MM (NIPAWILMA)
- JAK-STAT pathway inhibitors	Janus kinases (JAKs)	advanced solid tumors, NSCLC, lymphoma
- FAK inhibitors (FAKi)	Focal adhesion kinase (FAK)	CRC, NSCLC, combination ImT
2. Apoptosis modulators		
- BH3 mimetics, BCL-2/X_L_-inhibitors	Intrinsic cell death (NOXA, PUMA, BIM)	ALL (*Venetoclax*), n/f ^1^ (pre-clinical)
- SMAC mimetics	Inhibitor of apoptosis proteins (IAPs)	advanced solid tumors, NSCLC
- BCL-2 antisense oligonucleotides	Anti-apoptotic BCL-2 members	HM, MM (*Oblimersen*, withdrawn)
- p53 reactivation	Tumor suppressor p53, MDM2/4	Advanced cancers, lymphoma, AML
- Death receptor activation	Death receptors (FAS, TRAIL/APO-2)	MM (*DS-8273a*), NSCLC, CRC
- Autophagy inhibitors	Autophagy pathways	CRC, HCC, advanced solid tumors
3. DNA damage response inhibitors		
- PI3K/PIKK (ATM/ATR) inhibitors	ATM and ATR kinases	Advanced solid tumors, lymphoma, CNS
- Checkpoint kinase inhibitors	CHK1 kinase, WEE kinase	tumors (phase 0–II), ChT/RT combinations
- PARP inhibitors	poly(ADP-ribose) polymerase (PARP)	OC, combination therapies
4. Epigenetic drugs		
- 5-Aza-(2-deoxy-)cytidine	DNA-methyl-transferases (DNMT1-3)	HM (MDS), MM
- HDAC inhibitors (HDACi)	Histone deacetylases (HDAC1-10)	HM, lymphoma, N/SCLC, MM
- EZH2 inhibitors	Polycomb repressive complex (PRC) 2	Sarcoma, lymphoma, mesothelioma
- BET inhibitors	BET family of histone readers	Refractory HM (MDS, ALL), solid tumors
- DUB inhibitors	(Histone) deubiquitinases	n/f ^1^
- si/miRNAs	microRNAs, lncRNAs, tumor mRNAs	withdrawn, only biomarker studies
5. Telomerase inhibitors		
- hTR antagonists/competitors	hTR nucleotide (TERC)	solid tumors, withdrawn (*Imetelstat*)
- hTERT inhibitors (Ti)	Telomerase reverse transcriptase (TERT)	withdrawn
6. Redox drugs		
- Dimethylfumarate (DMF)	BCL2, PARP1, NFκB-NRF2-KEAP1-axis	Lymphoma (CTL), CNS (pre-clinical)
- Antioxidants (Vitamins, NAC, others)	EMT, cytotoxicity reduction	NSCLC, CRC, solid tumors, lymphoma
7. Metabolic drugs		
- Metformin, *Phenformin*	mTOR, TCA cycle	MM, BC, OC, PC, advanced solid tumors
- PDK inhibitors (Dichloroacetate)	Pyruvate dehydrogenase kinase, Glycolysis	CNS tumors (completed), SCC
8. Proteasome inhibitors		
- *Bortezomib*, PS-341	(Ubiquitin)-Proteasome pathway	Refractory solid tumors, HM
9. Transdifferentiation inducers		
- Retinoids (*ATRA*, *Bexarotene*)	Nuclear RAR and RXR receptors	HM, CNS, MM (pre-clinical)
- Unsaturated fatty acids	PPARγ, adipogenic differentiation pathways	CNS, MM (pre-clinical)
10. Immunomodulators		
- T cell receptor signaling enhancers	Ubiquitin ligases (CBL, ITCH, GRAIL, et al.)	n/f ^1^
	Adaptor proteins, kinases	
- Kynurenine pathway inhibitors	Indoleamine 2,3-dioxygenase (IDO1)	MM (KEYNOTE-252/ECHO-301, halted)
11. Others		
- Matrix metalloprotease inhibitors	MMP, COL3	Advanced solid tumors (completed)
- WNT/β-Catenin inhibitors	WNT ligand	WNT-driven tumors (MM, CRC, others)

^1^ n/f indicates that no ongoing clinical trial was found in public databases such as https://clinicaltrials.gov [19]. Abbreviations: acute lymphoblastic leukemia (ALL), central nervous system tumors (CNS), colorectal cancer (CRC), cutaneous T-cell lymphoma (CTL), hematological malignancies (HM), hepatocellular carcinoma (HCC), metastatic melanoma (MM), non-/small cell lung cancer (N/SCLC), ovarian carcinoma (OC), prostate carcinoma (PC), renal carcinoma (RC), squamous cell carcinoma (SCC), triple-negative/breast cancer (TN/BC), chemotherapy (ChT), immunotherapy (ImT), radiotherapy (RT).
